# Evaluation of color stability and bond strength of interim restorations fabricated by CAD/CAM and 3D printing: an in vitro study

**DOI:** 10.1186/s12903-026-08125-9

**Published:** 2026-04-11

**Authors:** Yasser M. Aly, Magued H. Fahmy, Nermeen Sweid

**Affiliations:** 1 Conservative Dentistry Department, Faculty of Dentistry, Alexandria University, 29 Elfardous street, Smouha, Alexandria Egypt; 2 Oral Rehabilitation Sciences Department, Faculty of Dentistry, Beirut Arab University, Beirut, Lebanon; 3 Oral Surgery Department, Faculty of Dentistry, Beirut Arab University, Beirut, Lebanon; 4 Prosthetic and Esthetic Dentistry, Faculty of Dentistry, Beirut Arab University, Beirut, Lebanon

**Keywords:** 3D printing, bond strength, CAD/CAM, color stability, interim restorations, PMMA, and surface treatments

## Abstract

**Background:**

Mechanical properties of interim restorations, as well as fabrication methods, are important for the integrity of restorations, especially for a long interim period. Interim restorations are subjected to occlusal loads; therefore, an adequate bond strength should be maintained until the final restoration is delivered. Color stability is also crucial in the esthetic zone throughout the long-term treatment phase. Therefore, both color stability and bond strength are dependent on the fabrication methods.

**Aim of Study:**

to evaluate the color stability and bond strength of 3-D printed and CAD/CAM milled interim restorations, and to compare between different groups regarding their color stability and bond strength.

**Materials and methods:**

A total of 72 interim PMMA specimens were fabricated in two distinct shapes: disc-shaped (8 mm in diameter × 2 mm in height) and square-shaped (10 mm × 10 mm × 2 mm thick). Half of the specimens (*n* = 36) were manufactured using CAD/CAM milling, while the remaining half (*n* = 36) were produced through 3D printing. Within each fabrication method, the specimens were categorized into two subgroups: Subgroup A, which consisted of 12 square-shaped specimens designated for color stability testing, and Subgroup B, comprising 24 disc-shaped specimens intended for shear bond strength testing. In Subgroup B, the discs were further divided based on surface treatment into three groups: surface roughening with a bur (*n* = 8), sandblasting (*n* = 8), and an untreated control group (*n* = 8). Color stability and bond strength measurements were recorded for all relevant specimens.

**Results:**

In both CAD/CAM milled and 3D printed groups, there was a significant difference in the color stability of the specimens over time (*p* < 0.05). They had high discoloration values with CAD/CAM specimens, with a mean of 13.50 (0.10), while 3D printed specimens had a mean of 14.31(0.10). Shear bond strength was also significantly different between the experimental groups with CAD/CAM milled specimens: the control group, 0.53 ± 0.33; the sandblasted group, 1.16 ± 0.25; and the roughened group, 1.00 ± 0.34. In contrast, the 3D printed specimens showed the following results: Control, 1.63 ± 0.90; Sandblasted, 1.44 ± 0.42; and roughened, 1.30 ± 0.43.

**Conclusion:**

CAD/CAM milled PMMA exhibited higher color stability compared to 3D printed PMMA, with coffee having a higher staining potential on PMMA than tea beverages. CAD/CAM shear bond strength was higher with surface treatment by aluminum oxide particles, while 3D printed PMMA has a higher shear bond without any surface treatment.

## Background

Interim, temporary, provisional, or transitional restorations are temporary dental prostheses used to enhance function, aesthetics, and stabilization for a limited time before being replaced by permanent or definitive prostheses [1]. Provisional restorations have to be used from the initial tooth preparation until the placement of the definitive prosthesis [2].

The requirements of a provisional restoration are essentially the same as for the definitive restoration, except for longevity and possibly sophistication of color [3]. Physicians should consider the type of materials [4], simplicity of fabrication and bonding [5], mechanical properties, and oral environmental conditions [6] when choosing long-term provisional restorative materials. Although all these purposes are important, the aesthetics of the provisional restoration is in many cases of prime importance to the patient, especially in cases where the provisional restorations are going to be used for a long period and in the aesthetic zone [7].

The advent of digital technology has revolutionized numerous areas of dentistry, including the restorative field. Traditional laboratory methods have been gradually supplanted by “digital workflow” processes [8].

Polymethyl methacrylate (PMMA) is a widely used material in prosthetic dentistry, particularly for long-term provisional crowns. With advancements in digital dentistry, polymethyl methacrylate (PMMA) can now be fabricated using computer-aided design/computer-aided manufacturing (CAD/CAM) through either subtractive milling of prefabricated blocks or additive three-dimensional (3D) printing. Milled CAD/CAM composites, polymerized under high temperature and pressure, offer improved mechanical properties and reduced water sorption compared to conventional materials. In contrast, 3D printing builds restorations layer by layer, and differences in these fabrication techniques may influence key clinical outcomes such as color stability and bond strength [9, 10].

Color stability is a crucial factor in the longevity of provisional crowns, as discoloration can compromise esthetics and patient satisfaction. PMMA restorations are exposed to various staining agents in the oral environment, including beverages like tea and coffee. The extent of discoloration is commonly assessed using the color difference (ΔE) measurement, which quantifies perceptible changes over time. The aesthetic aspect of dental restorations heavily relies on color harmony between artificial and natural teeth. This requirement extends to temporary restorations utilized in the dental restoration process, which must exhibit a quality level similar to that of natural dentition. These provisional restorations should also demonstrate resistance to staining from external colorants, such as those found in food and beverages.

Temporary crowns and fixed dental prostheses (FDPs) are frequently necessary to ensure long-term stability and protect teeth during ongoing treatments [11]. These provisional restorations must satisfy not only aesthetic and biological requirements but also mechanical needs, such as withstanding dislodging forces and functional loads [12]. Bond strength is another critical property, particularly for ensuring the durability of repairs or modifications in provisional restorations.

Differences in polymerization techniques and material composition between CAD/CAM and 3D-printed PMMA may influence their ability to maintain color stability and bond integrity over prolonged use. In oral conditions, these restorations are subjected to various forces: compressive force at the point of load application, and tensile and shear forces where the load is resisted. From a clinical perspective, evaluating shear bond strength is considered more appropriate than tensile testing because stress is distributed uniformly during shear tests. However, this method is sensitive to technique [13]. The ability of provisional restorations to remain securely in place for extended periods is crucial in prosthodontic treatment. Despite this, no research has examined the bond strength between these temporary restorations and tooth structure.

This study aimed to evaluate and compare the color stability and bond strength of PMMA fabricated using CAD/CAM and 3D printing techniques. Using Bego PMMA, the materials were subjected to staining in tea and coffee, and ΔE values were recorded to assess discoloration. Additionally, bond strength tests were conducted to determine the adhesive reliability of each fabrication method. The findings of this study provided insights into the clinical performance of digitally fabricated PMMA provisional crowns, helping guide material selection for long-term use. The null hypothesis of this study was that there would be no significant difference in the color stability and shear bond strength of interim PMMA specimens fabricated by CAD/CAM milling and 3D printing, regardless of the staining solutions or different surface treatments.

## Methods

This in vitro experimental study was conducted on 72 PMMA specimens fabricated by two different fabrication methods, CAD/CAM and 3D printing. Sample size was based on 80% study power and a 5% alpha error. Based on a comparison of independent means (effect size d = 2.13), the required sample size for color stability was calculated to be 5 specimens per group, increased to 6 to make up for laboratory processing problems. The total required sample size for color stability testing = number of groups × number per group = 6 × 4 = 24 specimens. Sample size was calculated using G*Power (Version 3.1.9.7) [14].

Based on a comparison of independent means (effect size d = 1.7), using the highest standard deviation for shear bond strength calculation to ensure adequate power, the required sample size was calculated to be 7 specimens per group, increased to 8 to make up for laboratory processing problems. The total required sample size for shear bond strength testing = number of groups × number per group = 6 × 8 = 48 specimens [15].

The materials in this study were selected based on usage and long-term verification in clinical practice. The two studied provisional restorative materials are manufactured by Bego, Germany. A baseline shade (A2) was used in all the study specimens. The materials VarseoSmile and PMMA Multicolor were obtained from the manufacturer as liquid resin for 3D printing and pre-polymerized blocks for milling.

### Specimen preparation and fabrication

A total of 72 PMMA specimens were fabricated and allocated to two main experimental groups: color stability testing (*n* = 24) and shear bond strength testing (*n* = 48). All specimens were digitally designed using 3D modeling software (Autodesk 3ds Max) and exported in STL format to ensure precision and reproducibility according to the fabrication method (milling or 3D printing).

### Color stability evaluation

**Fig. 1:**
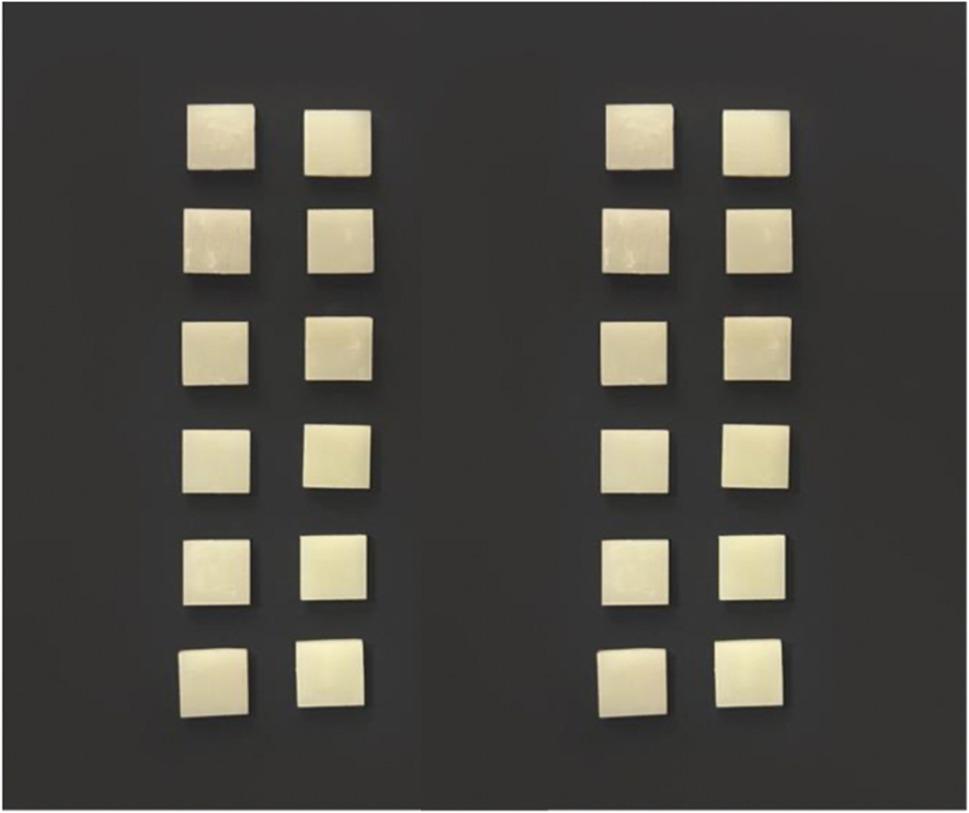
Square-shaped specimens for color stability measurement

A total of 24 square-shaped specimens (Fig. 1) were fabricated with a dimension of (10 mm x 10 mm x 2 mm), according to the International Organization of Standardization ISO 10,477;2004 for polymer-based crown and bridge material. Specimens were digitally designed using 3D modeling software (Autodesk, 3ds Max software) and exported as an STL file format [14].

12 square-shaped specimens were milled using (Ceramill Map 400; AmannGirrbach) from multicolor pre-polymerized PMMA blocks, and 12 square-shaped specimens were printed using 3D printing (Printer Halot Lite, Creality, Shenzhen, China) from VarseoSmile Temporary resin material. Each main group was divided into two subgroups (*n* = 6). One subgroup was immersed in a coffee solution and the other in a tea solution.

### Staining solutions

Two staining solutions were used to evaluate the color stability of PMMA, Coffee, and Tea. Coffee was prepared by mixing 8 g of coffee powder (Najjar, Turkish Coffee, Lebanon) with 300 cc of boiling water for 6 min. Black tea was prepared by placing one tea bag (Lipton, Unilever Korea Co., Ltd., Seoul, Korea) in 500 cc of boiling water for 15 min. The staining solutions were refreshed daily, and the experiment was carried out for 2 weeks.

### Finishing and polishing protocol

To ensure consistency across all groups, a standardized finishing and polishing protocol was followed. All specimens were cleaned using air jets, then surfaces were polished using silicon carbide abrasive paper, starting from 600-grit and progressing to 1200-grit, under constant water cooling to prevent overheating and ensure uniform surface quality. After that specimens were cleaned with 70% isopropyl alcohol for 1 min. Finally, this was followed by ultrasonic cleaning in distilled water for 3 min to remove any residual particles [16].

### Dimensional verification

A digital caliper was used to verify the thickness and dimensions of all specimens, ensuring standardization and measurement accuracy across groups.

### Assessment of color stability

#### Color measurement protocol

Color values were measured using a spectrophotometer (VITA Easyshade Compact) at three-time intervals. A baseline record was taken before specimen immersion, T1 after 7 days of immersion in staining solutions, and T2 after 14 days of immersion [17].

Color changes were quantified using the ΔE (Delta E) formula, which measures the color difference between two readings in the CIELAB color space. A spectrophotometer was used to record L*, a*, and b* values at baseline and after staining intervals. The ΔE value was then calculated to determine the degree of color change between CAD/CAM and 3D printed PMMA specimens over time [18].

The color space reference scale CIED2000 was used, and the spectrophotometer was calibrated using an automatic calibration block holder at 10 am in daylight conditions to determine the correct color of the specimens. Each reading was recorded three times at the center of the specimens, and a mean was calculated.

Using this equation:

$$\Delta \mathrm{E}_{00} \left[ \left(\frac{\Delta \mathrm{L}^{\prime}}{\mathrm{k}_{\mathrm{L}}\mathrm{S}_{\mathrm{L}}}\right)^{2} + \left(\frac{\Delta \mathrm{C}^{\prime}}{\mathrm{k}_{\mathrm{C}}\mathrm{S}_{\mathrm{C}}}\right)^{2} + \left(\frac{\Delta \mathrm{H}^{\prime}}{\mathrm{k}_{\mathrm{H}}\mathrm{S}_{\mathrm{H}}}\right)^{2} + \mathrm{R}_{\mathrm{T}}\left(\frac{\Delta \mathrm{C}^{\prime}}{\mathrm{k}_{\mathrm{C}}\mathrm{S}_{\mathrm{C}}}\right) \left(\frac{\Delta \mathrm{H}^{\prime}}{\mathrm{k}_{\mathrm{H}}\mathrm{S}_{\mathrm{H}}}\right)\right]^{\frac{1}{2}}$$

### Bond strength measurement

#### Specimen preparation for shear bond strength

**Fig. 2:**
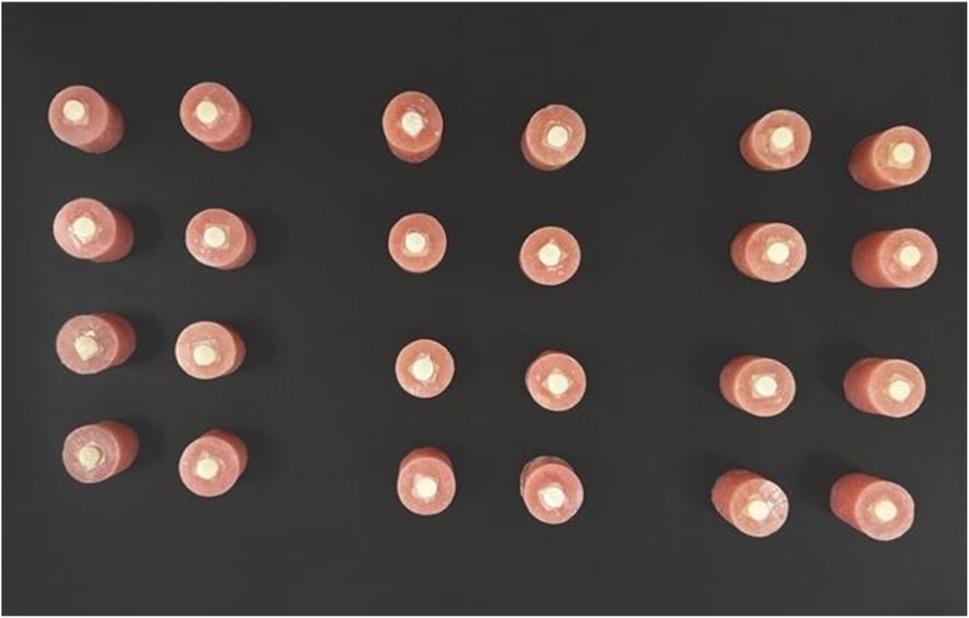
Acrylic resin blocks with composite embedded and disc shaped specimens cemented for testing shear bond strength

For measurement of shear bond strength, 24 discs with a dimension (8 mm in diameter x 2 mm in height) were prepared from each material milled and 3D printed (Fig. 2). The digitally designed geometry was designed using 3D modeling software (Autodesk, 3ds Max software) and then exported as an STL file format. 24 disc shaped specimens were milled using (Ceramill Map 400; AmannGirrbach) from multicolor pre-polymerized PMMA blocks, and 24 disc shaped specimens were printed using 3D printing (Printer Halot Lite, Creality, Shenzhen, China) from Varseosmile Temp resin material [12]. The same finishing and polishing protocols were followed as mentioned in the color stability testing.

#### Surface treatments

After finishing and polishing, each main group was subdivided into three subgroups (*n* = 8) according to the surface treatment employed.

Group 1 (Control group): 8 discs were left with no treatment.

Group 2 (sandblasted Group): 8 discs were sandblasted with 50 μm aluminum oxide particles using an airborne-particle abrasion device (Cobra, Renfert GmbH, Hilzinge, Germany) for 5 s at a standoff distance of 10 mm and a compressed air pressure of 276 Kpa.

Group 3 (Bur Roughened): 8 discs were surface roughened using a red-banded fine-grain-size diamond bur (TR-13 F). 

#### Specimens bonding

To bond these disc specimens to a tooth-like material, cube-shaped resin molds were printed by a 3D printer with a diameter of (10 mm x 10 mm x 3 mm). These molds were coated with a thin layer of separating material of petroleum jelly (Vaseline®; Unilever, London, UK) and then filled with composite resin (3 M. Espe Filtek Z250, Restorative Composite). Composite was applied in incremental layers and cured for complete polymerization (LED Light Bluephase® N, Ivoclar Vivadent, Schaan, Liechtenstein, with a wavelength range of 430–490 nm for 60 s, according to the manufacturer’s instructions) to fill the 3 mm thick resin mold. The composite cubes were then cleaned and polished.

The polymerized composite cubes were embedded in acrylic cylinders (customized acrylic cylinder mold). Acrylic cylinders were made by self-curing acrylic material (HUGE Dental, Singapore) with a mixing ratio of 1 ml liquid; to 2.2 g powder, mixing time 0.5 min and left to set for 30 min curing time. After that, the composite cubes were spot etched (in the center) for 15 s by applying a 37% phosphoric acid etchant (Meta Etchnat, Meta Biomed, Korea), followed by rinsing and drying. A bonding agent (3 M ESPE Single Bond Universal, 41282) is applied using a micro brush for 15 s and then light cured for 20 s with LED Light Bluephase^®^ N, (Ivoclar Vivadent, Schaan, Liechtenstein) following manufacturer’s instructions. Cementation of the surface treated PMMA discs to the composite cubes was carried out using 3 M ESPE RelyX Universal Resin Dental Cement, using a static loading cementation machine (0.2 kg load) to ensure uniform load during bonding procedure [15].

**Fig. 3:**
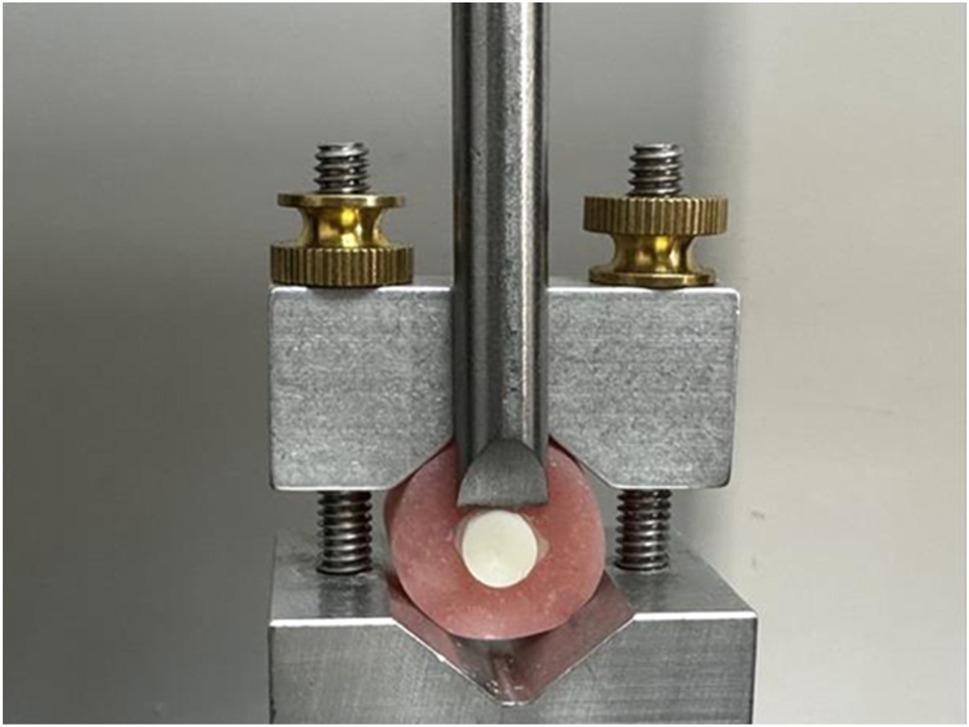
Frontal View of SBS measurement by Universal Testing Machine

Shear bond strength was measured by applying a static load through a universal testing machine (YLE, GmbH, Helsinki, Finland) (Fig. 3). The bonded disc specimen was positioned in the machine’s lower jaw so that it was parallel to the direction of the shear force. At a crosshead speed of 0.5 mm/min, a compressive loading was applied.

The debonding force (the force at which the specimen debonded) was displayed in Newton (N) on the connected computer. The shear bond strength (MPa) was calculated by dividing the fracture load (N) by the area of the bonded interface (mm^²^).

SBS = F/A 

F: Fracture Load in Mpa

A: Area of bonded interface in mm^2^

**Fig. 4:**
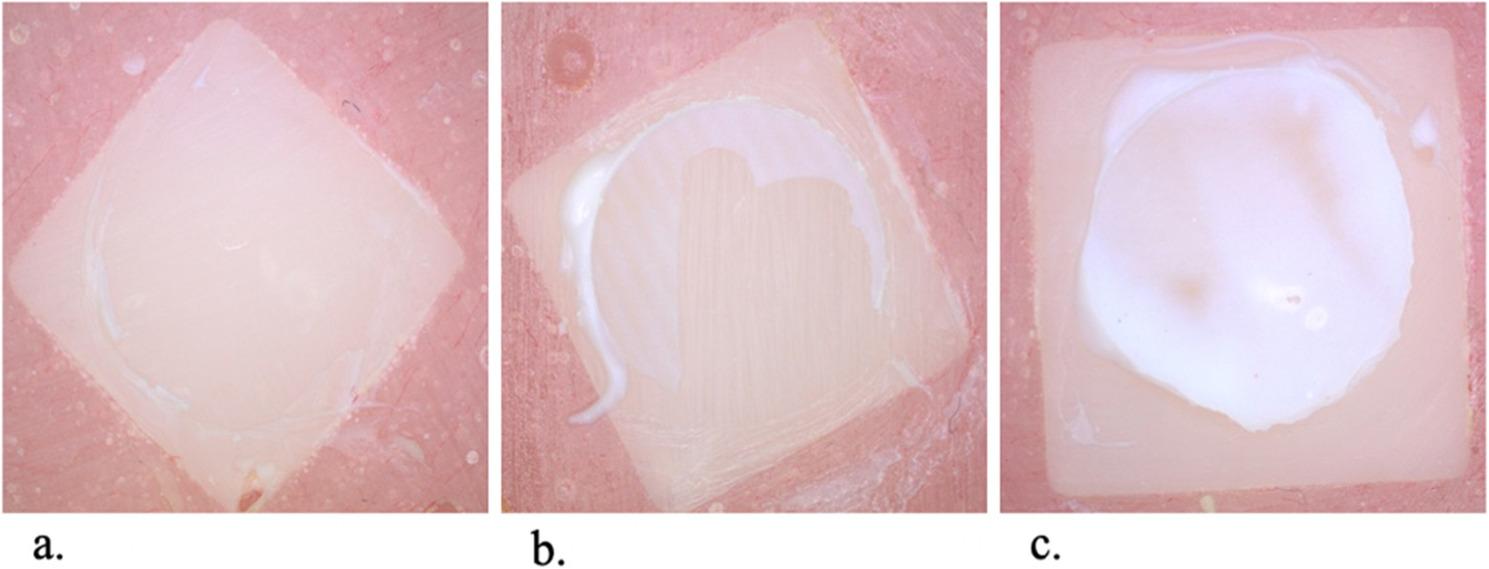
**a** Showing adhesive failure mode. **b** Showing mixed failure mode. **c** Showing cohesive failure mode

All specimens were observed for three types of failure modes: adhesive, cohesive, and mixed. Using a stereomicroscope, failure mode was defined as adhesive when more than 75% of the core build-up surface was visible. The cohesive failure mode was defined when more than 75% of the core build-up surface was covered with resin or the fracture was inside the core build-up material. All other cases were classified as having a mixed failure mode (Fig.4).

### Statistical analysis

Data were collected and tested for normality using SPSS software. According to the results of Shapiro-Wilk test, it was confirmed that all data were normally distributed, so means and standard deviation (SD) were calculated, and parametric tests were used. This ensured the appropriate application of parametric statistical tests. Comparisons between the two study groups were performed using an independent samples t-test, with calculation of mean differences and 95% confidence intervals (CI)s. Repeated measures ANOVA was employed to assess changes over time within each group, followed by post-hoc Bonferroni corrections for pairwise comparisons. Two-way ANOVA was used to assess the effect of groups and subgroups. Statistical significance was set at *p-*value < 0.05 for all analyses. Data were analyzed using IBM SPSS Statistics for Windows, Version 26.0 (IBM Corp., Armonk, NY, USA).

## Results

Data were collected and tested for normality using IBM SPSS software for Windows (Version 26.0). According to the results of Shapiro-Wilk test, it was confirmed that all data were normally distributed. This ensured the appropriate application of parametric statistical tests.

### Color stability

The results in Table 1 compared the color change (∆E00) between CAD/CAM milled and 3D printed specimens at different timepoints (T1, and T2).

**Table 1:** Comparison of color change (∆E_00_) between the two study groups at different timepoints

		CAD/CAM milled	3D printed	Difference (95% CI)	T test	df	*p* value 1
		Mean ± SD					
C- Specimens	**T1**	13.71 ± 0.26	14.98 ± 0.40	-1.27 (-1.70, -0.84)	**6.55**	**10**	< 0.001*
	**T2**	12.51 ± 0.67	13.19 ± 0.75	-0.68 (-1.59, 0.24)	1.65	10	0.13
	**F test** **df** ***P*** **value 2**	37.16 2 0.001*	182.84 2 < 0.001*				
T- Specimens	T1	13.31 ± 0.32	13.87 ± 0.30	-0.56 (-0.96, -0.15)	3.07	10	0.01*
	**T2**	12.57 ± 0.71	11.91 ± 0.36	0.66 (-0.07, 1.38)	2.02	10	0.10
	**F test** **df** ***P*** **value 2**	24.88 2 0.001*	271.17 2 < 0.001*				
Overall	**T1**	13.51 ± 0.35	14.42 ± 0.67	-0.91 (-1.37, -0.45)	4.17	22	0.001*
	**T2**	12.54 ± 0.66	12.55 ± 0.87	-0.01 (-0.66, 0.64)	0.03	22	0.98
	**F test** **df** ***P*** **value 2**	58.32 2 < 0.001*	137.46 2 < 0.001*				

*SD* Standard Deviation, *CI* Confidence Interval

*statistically significant at *P*-value < 0.05

*P* value 1: Comparison between the two groups using independent samples t-test

*P* value 2: Comparison between different timepoints within each group using repeated measures ANOVA

Coffee Specimens (C-Specimens) at the initial timepoint (T1), the mean color change was significantly higher for the 3D printed group (14.98 ± 0.40) compared to the CAD/CAM milled group (13.71 ± 0.26), with a statistically significant difference of -1.27 (*p* < 0.001). By T2, although the mean difference between the two groups remained, it was no longer statistically significant (*p* = 0.13).

The repeated measures ANOVA results (*p* Value 2) indicate significant changes in color (∆E00) over time within both the CAD/CAM milled group (*p* = 0.001) and the 3D printed group (*p* < 0.001). These findings suggest that while the 3D printed specimens generally exhibited greater color changes than CAD/CAM milled specimens, the degree of change decreased over time, reducing the statistical significance by T2. This highlighted the temporal dynamics (pattern that occurs over time) of color stability in the two materials.

Table 1 also compared the color change (∆E00) between CAD/CAM milled and 3D printed specimens for Tea Specimens (T- Specimens) at different time points (T1, and T2).

At T1, the 3D printed group (13.87 ± 0.30) exhibited a significantly greater color change than the CAD/CAM milled group (13.31 ± 0.32), though the difference was smaller (*p* = 0.01).

By T2, the trend reversed slightly, with the CAD/CAM milled group (12.57 ± 0.71) showing a marginally higher mean color change compared to the 3D printed group (11.91 ± 0.36). However, this difference was not statistically significant (*p* = 0.10).

The repeated measures ANOVA (*p* Value 2) revealed significant changes in color over time within both groups, with *p* = 0.001 for the CAD/CAM milled group and *p* < 0.001 for the 3D printed group.

These findings indicated that while the 3D printed specimens initially demonstrated greater color change, the trend diminished over time, suggesting a potential stabilization of color properties in the 3D printed materials by T2.

### Shear bond strength

Table 2 provided a comparison of shear bond strength (MPa) between CAD/CAM milled and 3D printed specimens across different subgroups (Control, Sandblasted, Roughened) and overall results.

**Table 2:** Comparison of Shear Bond Strength (MPa) Between the Study Groups

	CAD/CAM milled	3D printed	Difference (95% CI)	T test	df	*P* value 1
	Mean ± SD					
Control	0.53 ± 0.33 **a**	1.63 ± 0.90	-1.10 (-1.83, -0.37)	3.24	14	0.006*
Sandblasted	1.16 ± 0.25 **b**	1.44 ± 0.42	-0.28 (-0.65, 0.09)	1.60	14	0.13
Roughened	1.00 ± 0.34 **b**	1.30 ± 0.43	-0.30 (-0.72, 0.11)	1.56	14	0.14
Overall	0.90 ± 0.40	1.46 ± 0.61	-0.56 (-0.86, -0.26)	3.73	46	0.001*
F test df P value	8.92 2 0.002*	0.55 2 0.58				

*SD* Standard Deviation, *CI* Confidence Interval, *df* degree of freedom

*Statistically significant at *p*-value < 0.05

*p* value 1: Comparison between the two groups using independent samples t-test

*p* value 2: Comparison between different subgroups within each group using one-way ANOVA (F)

a, b: different letters denote significant differences between subgroups using Bonferroni correction

Control Subgroup: The shear bond strength was significantly lower in the CAD/CAM milled group (0.53 ± 0.33 MPa) compared to the 3D printed group (1.63 ± 0.90 MPa), with a difference of -1.10 MPa (95% CI: -1.83 to -0.37, *p* = 0.006*). Sandblasted Subgroup: While the 3D printed group (1.44 ± 0.42 MPa) had a slightly higher mean shear bond strength than the CAD/CAM milled group (1.16 ± 0.25 MPa), the difference (-0.28 MPa; 95% CI: -0.65 to 0.09) was not statistically significant (*p* = 0.13). This suggests a comparable performance between the two groups after sandblasting treatment. Roughened Subgroup: Similarly, the 3D printed specimens (1.30 ± 0.43 MPa) demonstrated a marginally higher mean shear bond strength than the CAD/CAM milled specimens (1.00 ± 0.34 MPa), but the difference (-0.30 MPa; 95% CI: -0.72 to 0.11) was not statistically significant (*p* = 0.14).

Overall Comparison: Across all subgroups, the 3D printed specimens (1.46 ± 0.61 MPa) exhibited significantly higher overall shear bond strength compared to the CAD/CAM milled specimens (0.90 ± 0.40 MPa), with a difference of -0.56 MPa (95% CI: -0.86 to -0.26, *p* = 0.001*). *P* Value 2: The differences between subgroups within the CAD/CAM milled group were statistically significant (*p* = 0.002*), while no significant differences were observed within the 3D printed group (*p* = 0.58).

Table 3 presented the post-hoc comparisons of shear bond strength between different subgroups within the CAD/CAM milled group, highlighting the effects of surface treatments. The sandblasted subgroup exhibited significantly higher shear bond strength compared to the control subgroup, with a p-value of 0.002, indicating that sandblasting substantially improves bonding performance. Similarly, the roughened subgroup showed significantly higher shear bond strength compared to the control subgroup, with a *p*-value of 0.02, demonstrating the effectiveness of roughening as a treatment method. However, no significant difference in shear bond strength was observed between the sandblasted and roughened subgroups, with a p-value of 0.92, suggesting that both treatments result in comparable performance. These findings underscore the role of surface treatments in enhancing shear bond strength for CAD/CAM milled materials, while showing that sandblasting and roughening produce similar outcomes.

**Table 3:** post-hoc comparisons of shear bond strength between different subgroups within the CAD/CAM milled group

Group	Subgroup	Compared to	*p* value
CAD/CAM milled	Control	**Sandblasted**	0.002*
		**Roughened**	0.02*
	Sandblasted	**Roughened**	0.92

*Statistically significant using Bonferroni correction

### Mode of failure

A total of 48 disc shaped specimens were evaluated for failure mode distribution after shear bond testing. The CAD/CAM milled group showed predominantly cohesive failures (66.7%), followed by mixed failures (25.0%) and adhesive failures (8.3%). In contrast, the 3D-printed group exhibited primarily adhesive failures (66.7%), with mixed failures accounting for 25.0% and cohesive failures comprising only 8.3%, as shown in Table 4.

**Table 4:** Distribution of Failure Modes by Fabrication Method

Failure Mode	CAD/CAM (*n* = 24)	%	3D Printed (*n* = 24)	%
Adhesive	2	8.3%	16	66.7%
Cohesive	16	66.7%	2	8.3%
Mixed	6	25.0%	6	25.0%
Total	24	100%	24	100%

A chi-square test of independence revealed a statistically significant association between the type of fabrication method and the failure mode observed (χ² = 21.78, *p* < 0.001). This suggests that the mode of failure is significantly influenced by the method of PMMA fabrication.

## Discussion

Digital dentistry has evolved over the past decades and gained popularity because it offers many advantages over conventional techniques. The additive and subtractive digital workflow has efficiently allowed the fabrication of precise permanent and provisional restorations. In addition to the importance of permanent restorations, provisional restoration is a crucial phase in complex cases and long-term treatments.

In this study, CAD/CAM and 3D printing PMMA resin were immersed in coffee and tea beverages for 14 days. As a result, it was observed that 3D printing PMMA resin had lower color stability than all CAD/CAM PMMA resins. Furthermore, coffee caused more discoloration than the tea beverage in the tested specimens. Therefore, the null hypothesis that there is no difference in color stability between CAD/CAM and 3D printed PMMA material and that no difference in discoloration according to staining solution or storage time was rejected.

For color stability, the specimens were immersed in the staining solutions (Tea and Coffee) for 14 days, the solutions were refreshed daily, and the specimens were only immersed in distilled water without any surface cleaning or brushing. Before immersion, baseline measurements were taken, followed by T1 and T2 measurements taken after 7 and 14 days, respectively. Data were collected by a spectrophotometer (Vita EasyShade) for accurate, reliable, and objective data, against a grey background in order to enhance visibility and reduce background reflection [19]. In addition, as discussed in previous studies [20], the thickness of the material affects color stability; therefore, in this study, a thickness of 2 mm was used for standardization. The use of the CIED2000 color difference, since it reflects the color difference between tooth colors more accurately than the CIELab formula [18].

Various ΔE thresholds have been discussed in the literature, with a precision and acceptability range of 3.7 to 6.8 [21]. However, all ΔE values in this study exceeded the clinically acceptable thresholds. ΔE thresholds differed according to the material, staining solution, and storage time. First, it was obvious that 3D printing PMMA resin exhibited greater discoloration than CAD/CAM PMMA material in all groups. This suggests that 3D printing has lower color stability than CAD/CAM material, which is due to the fabrication process of 3D printing layer by layer, leading to weakness in the interface of the layers, and high-water sorption; also, incomplete post-curing may reduce the color stability of the material.

However, CAD/CAM had higher color stability than 3D printing resin. This is due to the dense nature of the CAD/CAM pre-polymerized blocks, higher crosslinking material, and high polymerization rate, which led to a more color-stable surface and made the material less prone to discoloration.

ΔE values in our study were similar to other studies, where values exceeded acceptable thresholds, and were much higher, ranging between 12 and 15 after 14 days of immersion. Sham et al. [22] found that ΔE values for methyl methacrylate were 10.3, and coffee caused more discoloration than other tested beverages.

Among the two used staining solutions, coffee caused the most color discoloration in both tested materials over the 14 days of storage time. A similar study was done by Penate et al., which compared color stability between CAD/CAM and conventional provisional material after immersion in six staining solutions. The results showed that coffee caused more staining in the material than black tea, that was contributed to the fact that the lower polarity of the coffee solution helps in more penetration of the pigments into the organic phase because of adsorption and absorption process, whereas high polarity solutions such as tea cannot penetrate easily into the provisional material, thus pigments can be easily removed by tooth brushing.

Findings of this study were in agreement with a previous article by Shin et al., [23], who found that 3D printed PMMA exhibited more discoloration when immersed in staining solutions (coffee, curry, and grapefruit) for 30 days than CAD/CAM milled PMMA. In another recent study, Gruber et al. [24] evaluated color change for heat polymerized resin, CAD/CAM PMMA resin, and 3D printed resin. The results showed no significant color change between heat polymerized resin and CAD/CAM resin; however, 3D resin showed higher color change and lower color stability.

A study by Stavros et al. [25] examined long-term color stability in provisional restorations. Specimens were measured at 1 day, 7 days, and 30 days after immersion; all ΔE* values were too high, which is in agreement with the results of our study. ΔE values ranged between 4 and 12 after 7 days, and these values continued to increase after 30 days of immersion, where values ranged between 5 and 15.

Also in this study, three different surface treatment methods were studied in order to see if there is any significant difference on PMMA bond strength fabricated by CAD/CAM and 3D printing.

The results of SBS values in the three different groups, SBS in the control group was significantly lower in the CAD/CAM milled group, with a value of 0.53 *±* 0.33 MPa, compared to the 3D printed group, 1.63 *±* 0.90 MPa. This indicates a notable advantage for 3D printed material over CAD/CAM milled PMMA. While in the Sandblasted group, the 3D printed group had a slightly higher SBS value, 1.44 *±* 0.42 MPa, over the CAD/CAM milled group with an SBS value of 1.16 *±* 0.25 MPa. The difference was not statistically significant, which suggests a similar performance between the two groups after sandblasting. However, in the bur roughened subgroup, 3D printed specimens exhibited a higher SBS value, 1.46 *±* 0.43 MPa, over CAD/CAM milled specimens with an SBS value of 1.00 *±* 0.34 MPa, but the difference was not statistically significant.

The overall comparison difference between the CAD/CAM milled and 3D printed groups was statistically significant, with a p-value of 0.001. These results indicate that 3D printed specimens demonstrate superior shear bond strength compared to CAD/CAM milled specimens, highlighting the influence of manufacturing technique on bonding performance.

These results may be explained by the fact that bonding to acrylic is difficult because PMMA is chemically inert, highly polymerized, and lacks reactive sites for bonding agents. Its smooth and hydrophobic surface limits adhesive wetting and mechanical retention. PMMA, especially in CAD/CAM fully polymerized blocks, has minimal residual monomer, which means poor chemical bonding potential. Therefore, roughening and chemical primers are needed, but the bond is mostly mechanical and can degrade over time [26].

Adequate surface treatments should be carefully selected and utilized for each temporary restoration system due to the differences in chemical compositions of the temporary crown and FDP materials. For the selection of the optimal surface treatment for every clinical situation, it is critical to know the bond strengths resulting from different surface treatments [12].

This study tested the null hypothesis that fabrication method (CAD/CAM milling vs. 3D printing) would not significantly affect the color stability or shear bond strength of interim PMMA specimens, irrespective of staining solutions or surface treatment. The results partially rejected this hypothesis, as differences were observed in color stability and shear bond strength results.

## Conclusions

Within the limitations of this in vitro study, the following was concluded:

- The findings of our study revealed that CAD/CAM milled PMMA exhibited better color stability in long-term use over 3D printed PMMA.
- Coffee had a higher discoloration ability on PMMA specimens in both groups than black tea.
- Concerning the shear bond strength, a non-significant difference occurred between surface-treated 3D printed specimens by sandblasting and bur roughening.
- The 3D printed specimens control group without any surface treatment yielded the highest shear bond strength among all study groups.
- CAD/CAM milled specimens had a higher shear bond in sandblasted specimens.

## Data Availability

The database used and/or analyzed during this study are available from the corresponding author on reasonable request.

## Abbreviations

3D: Three Dimensional
CAD/CAM: Computer Aided Design/ Computer Aided Manufacturing
CI: Confidence Interval
CIE: International Commission on Illumination
CIELAB: International Commission on Illumination, L*Lightness a* red to green coordinate, b* blue to yellow coordinate
C-Specimens: Coffee Specimens
DLP: Digital Light Processing
MPa: Mega Pascale
PMMA: Poly Methyl Methacrylate
SBS: Shear Bond Strength
SLA: Stereo Lithography Apparatus
STL: Standard Tessellation Language
T-Specimens: Tea Specimens
UV: Ultra Violet
